# A Rare Variant of Pancreatic Sarcoidosis: Diagnostic Challenge

**DOI:** 10.5005/jp-journals-10018-1149

**Published:** 2016-07-09

**Authors:** Taran Khangura, Gias Uddin, Albert Davies, John Keating

**Affiliations:** 1 Department of Gastroenterology and Hepatology, Furness General Hospital, University Hospitals of Morecambe Bay NHS Foundation Trust, Barrow in Furness, Cumbria, LA14 4LF, United Kingdom

**Keywords:** Diagnosis, Noncaseating granuloma, Pancreatic carcinoma, Pancreatic sarcoidosis, Sarcoidosis.

## Abstract

Pancreatic sarcoidosis is one of the variant of systemic sarcoidosis which is extremely rare in literature and opined as an enigma. Hence, its diagnosis is very challenging as its presentation mimics pancreatic carcinoma. In view of a better understanding, we are presenting a case of pancreatic sarcoidosis to emphasize the above. To clearly distinguish sarcoidosis from malignancy, repeated computed tomography scans, Positron emission tomography scans, fine needle aspiration cytology, endoscopic retrograde cholangio-pancreatography supplemented with histology and relevant blood tests proved it to be a pancreatic sarcoidosis then a malignancy. Over the course, concrete evidence was divulged from lymph node biopsy and histology in clinching the diagnosis which later transformed into a malignancy. Therefore, we concluded it is a rare variant of pancreatic sarcoidosis and careful serial investigations include repeated imaging, histology and blood tests are essential to establish and most importantly differentiate the diagnosis.

**How to cite this article:**

Khangura T, Uddin G, Davies A, Keating J. A Rare Variant of Pancreatic Sarcoidosis: Diagnostic Challenge. Euroasian J Hepato-Gastroenterol 2015;5(2):118-121.

## INTRODUCTION

Primary sarcoidosis of the pancreas is extremely rare. Clinical presentation may mimic pancreatic cancer. We present an unusual case of a 61-year-old lady who presented with abdominal pain and obstructive jaundice. Computed tomography (CT) scan showed mass in the pancreatic head. She was diagnosed to have inoperable cancer. Her diagnosis was confirmed with a positron emission tomography (PET) scan which revealed fluoro-deoxyglucose (FDG)-avid mediastinal and cervical lymph nodes. Upon return of biopsy reports, however, histology was reported to be consistent with sarcoidosis and therefore it was presumed that she also had sarcoidosis of the pancreas. After a few months she became unwell and CT scan revealed metastatic pancreatic malignancy.

## BACKGROUND

Sarcoidosis is a noncaseating granulomatous disease of unknown etiology. It affects mainly lungs (90%) and lymph nodes (75%), eyes (25%) and skin (25%). However, it can affect almost any organ in the body.^1,2^ Involvement of liver and pancreas is rare. About 1 to 5% of patients with systemic sarcoidosis have pancreatic sarcoidosis in autopsy studies.^3-7^ Pancreatic sarcoidosis is a rare variant of systemic sarcoidosis. The modern history of sarcoidosis, an enigmatic multisystem disease, goes back to 1899, when the pioneering Norwegian dermatologist Caesar Boeck coined the term to describe skin nodules characterized by compact, sharply defined foci of ‘epithelioid cells with large pale nuclei and also a few giant cells’.^8^ Thinking this resembled sarcoma, he called the condition ‘multiple benign sarcoid of the skin’. It is important that in addition to microscopic involvement of various organ systems, sarcoidosis can produce bulky mass-like granulomatous tissue that mimics malignant disease radiographically.^9^

## CASE REPORT

**Fig. 1:**
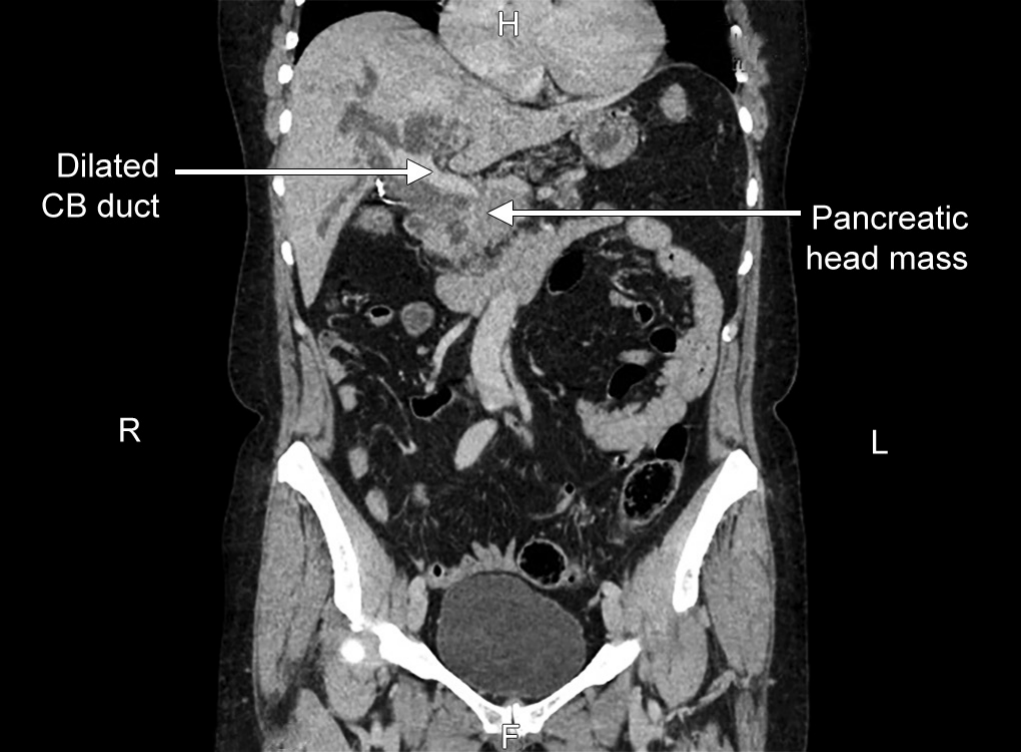
Computed tomography scan of abdomen showing pancreatic head mass

**Fig. 2:**
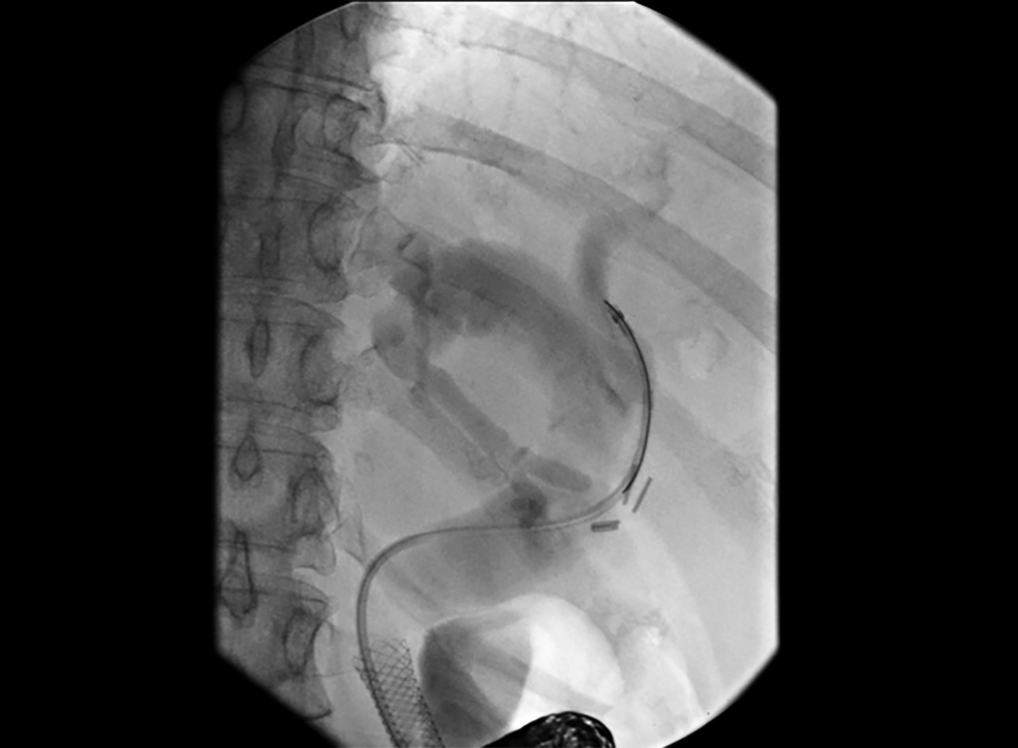
Endoscopic retrograde cholangio-pancreatography (ERCP) image of stent

A 61-year-old lady first presented initially in February 2013 with abdominal pain and then developed obstructive jaundice. The data of biochemical tests are shown in Table 1. Radiographic imaging at that time revealed a mass in the head of her pancreas (Fig. 1). A metallic biliary stent was inserted to relieve obstructive jaundice (Fig. 2). Unfortunately, she became septic following the procedure delaying her stay in hospital for several weeks.

**Table 1:** **Table 1:** Biochemical findings during the course of illness in hospital including routine blood tests, tumor markers and marker for sarcoidosis

*Serum tests*		*26.04.13*		*16.04.14*		*17.04.14*		*17.05.14*		*04.06.14*		*12.08.14*	
LFT		Initial		Pre-stent		Post-stent		—		Review		Final	
• ALT (IU/1)		459		42		24		—		16		30	
• ALP (IU/l)		646		701		514		—		267		596	
• GGT (IU/l)		1145		499		430		—		223		616	
• Total protein (gm/l)		73		58		66		—		69		72	
• Albumin (gm/l)		45		27		32		—		36		37	
• Globulin (gm/l)		28		31		34		—		33		35	
• Bilirubin (umol/l)		155		55		51		—		12		10	
CRP		6.7		87.3		28.6		—		9.2		92.7	
CA 19-9		26.8		1161		66.2		—		87.6		85.9	
FBC													
• Hb (g/l)		130		70		107**		—		101		93	
• WBC (10*9/l)		5.4		6.4		9.3		—		5.4		7.5	
• Platelet (10*9/l)		188		167		120		—		161		153	
• Neutrophil (10*9/l)		3.6		4.6		6.3				3.9		5.8	
Serum ACE levels IU/l								33		60			

**Post-transfusion Hemoglobin; LFT: Liver function tests, ALT: Alanine aminotransferase; ALP: Alkaline phosphatase; GGT: Gamma glutamyl transferase; CRP: C-reactive protein; Ca 19-9: Carbohydrate antigen 19-9; FBC: Full blood count; Hb: hemoglobin; WBC: White blood cells; ACE: Angiotensin converting enzyme (reference range: U/L 8-59)

**Fig. 3:**
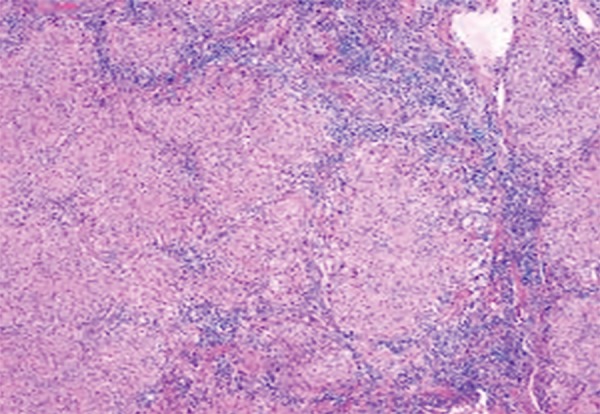
Pancreatic sarcoidosis in lymph node

In order to clarify her diagnosis, she was referred to a specialist center for an endoscopic ultrasound scan (EUS) and fine needle aspiration (FNA). This was performed on three or four occasions, however the cytology did not reveal any malignancy but inflammatory cells were identified on each occasion. In view of the uncertainty of the diagnosis and lack of progression on repeated CT scans, she went onto having a PET CT scan which revealed FDG-avid mediastinal and cervical lymph nodes. These nodes were biopsied for further analysis. Lymph node biopsy showed non-caseating granuloma (Fig. 3).

### Differential Diagnosis

Given the patient’s history and initial findings it was suggested to be pancreatic cancer without being formally diagnosed. However, the histology of the lymph nodes was reported to be consistent with sarcoidosis and therefore it was presumed she also had sarcoidosis of the pancreas (Fig. 3). Her family history revealed her mother had bowel cancer and father had stomach cancer. Her past surgical history entailed that this lady underwent a hemithyroidectomy for a thyroid nodule performed some years earlier and histology was benign. Infectious causes and lymphoma are possible differentials.

### Progression and Treatment

In January 2014, she became unwell again and was admitted to hospital with pleurisy and sepsis. Subsequently, a further biliary stent change was performed. In March 2014, she was readmitted with another episode of sepsis possibly due to cholangitis. She was in hospital for 5 weeks during which her plastic biliary stent was changed for a self-expanding metal stent in April 2014. She was treated with enoxaparin for a deep vein thrombosis (DVT) she developed in her left leg.

**Fig. 4:**
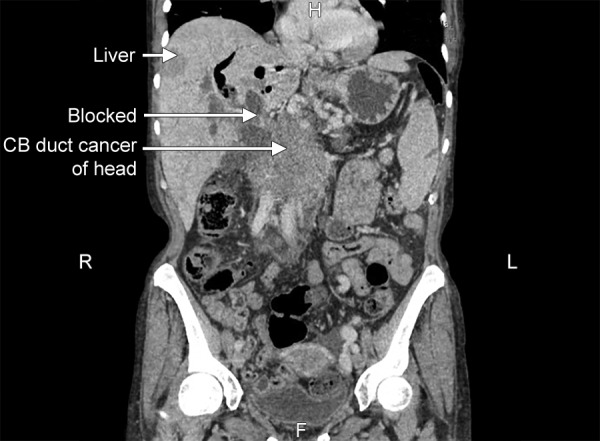
Computed tomography scan of abdomen showing pancreatic cancer with metastasis

### Outcome and Follow-up

Once pancreatic sarcoidosis was confirmed on biopsy, she was started with systemic corticosteroids which provided symptomatic relief. Her medication included a proton pump inhibitor, diarrhea was managed with pan-creatin 10,000 units increased to 25,000 units for snacks and 50,000 units for meals, analgesics for intermittent abdominal pain and recently commenced ursodeoxy-cholic acid to try and prevent her stent from silting up in the future. After a course of steroids and conservative therapy, the patient became well and managed to go home in April 2014.

However, on a routine outpatient clinic follow-up in August 2014, she was found to be lethargic with weight loss, anemic and signs of obstructive jaundice. At this stage, immediate hospital admission was deemed necessary and symptomatic treatment with blood transfusion, nourishment and recommencement of systemic steroids was initiated. A repeat CT of the chest, abdomen and pelvis showed total distortion of the pancreatic tissue with irregular margin along with multiple cystic deposits were at this stage consistent with metastatic disease (Fig. 4). The patient was not willing to undergo a further biopsy, and in view of her continuing deterioration of her general condition was referred to palliative care.

## DISCUSSION

Sarcoidosis is a systemic inflammatory disease of unknown origin characterized by the formation of non-caseating granulomas.^9^ Virtually any organ system may be involved. Although involvement of abdominal viscera is less frequent than pulmonary and mediastinal disease, when it occurs, it may mimic more common infectious or neoplastic conditions and result in unnecessary morbidity.^10-12^ In 2006, a literature review by Caceres et al demonstrated 25 cases of surgically proven pancreatic sarcoidosis with 12 presenting as a pancreatic head mass and only 4 being symptomatic.^13^

Currently, there is no consensus on the best imaging modality for pancreatic evaluation. However, endo-sonographic imaging may be helpful. A prospective study of 156 patients in 2011 by Brimiene et al concluded that ultrasonography is the superior initial diagnostic technique for differentiating chronic pancreatitis and adenocarcinoma.^14^

Endoscopic retrograde cholangio-pancreatography (ERCP) with endosonographic view and FNA from pancreas will give a confirmatory diagnosis from histopatho-logy. Computed tomography scan, magnetic resonance imaging (MRI) scan, ultrasound (US) scan are helpful in differentiating other causes of similar presentations, such as pancreatic cancer, intraductal papillary mucinous neoplasm (IPMN) and cysts. Tumor markers also give an indication about the possible origin of cancer. However, biopsy and histopathology from the affected organ, such as the pancreas or lymph nodes remains the gold standard modality.

## CONCLUSION

Malignant pancreatic tumor and pancreatic sarcoido-sis could be asymptomatic but may present in similar clusters of signs and symptoms, such as; obstructive jaundice, upper abdominal pain, nausea with stomach fullness, offensive flatulence and unintentional weight loss. Blood tests, such as abnormal LFTs (markedly raised bilirubin and elevated transaminases) could give a clue on obstructive jaundice; raised Ca19-9 can be found in both pancreatic tumors and to a lesser extent in pancreatic sarcoidosis; very high serum ACE can present in any form of sarcoidosis. Ultrasound, CT scan, MRI could be helpful tools but ERCP followed by FNAC remains gold standard for a confirmatory diagnosis. We concluded from this case report, that this is a rare variant of likely primary pancreatic sarcoidosis which transformed into malignancy involving pancreas itself, involving medias-tinal and cervical lymph nodes and liver.
